# Potential survival benefits of open over laparoscopic radical gastrectomy for gastric cancer patients beyond three years after surgery: result from multicenter in-depth analysis based on propensity matching

**DOI:** 10.1007/s00464-021-08430-0

**Published:** 2021-06-03

**Authors:** Ze-Ning Huang, YuBin Ma, Qi-Yue Chen, Chao-Hui Zheng, Ping Li, Jian-Wei Xie, Jia-Bin Wang, Jian-Xian Lin, Jun Lu, Long-Long Cao, Mi Lin, Ru-Hong Tu, Ju-Li Lin, Hua-Long Zheng, Chang-Ming Huang

**Affiliations:** 1 Department of Gastric Surgery, Fujian Medical University Union Hospital, Fuzhou, China; 2 Department of General Surgery, Fujian Medical University Union Hospital, Fuzhou, China; 3 Key Laboratory of Ministry of Education of Gastrointestinal Cancer, Department of Gastric Surgery, Fujian Medical University Union Hospital, No. 29 Xinquan Road, Fuzhou, 350001 Fujian China; 4 Department of Gastrointestinal Surgery, Qinghai University Affiliated Hospital, Xining, China

**Keywords:** Survival benefits, ADGC, LDG, Muticenter, Propensity matching

## Abstract

**Background:**

The oncologic efficacy of laparoscopic versus open surgery for advanced distal gastric cancer (ADGC) beyond 3 years after surgery remain obscure.

**Methods:**

A total of 1256 patients with ADGC at two teaching institutions in China from April 2007 to December 2014 were enrolled. The general data of the two groups were identified to enable rigorous estimation of propensity scores. Restricted mean survival time (RMST) and Landmark analysis was used to compare survival.

**Results:**

After matching 461 patients each in the open distal gastrectomy (ODG) and laparoscopic distal gastrectomy (LDG) groups, they were included into analysis. The 3- and 5-year overall survival (OS) and disease-free survival were comparable in two groups. RMST-stratified analysis showed that the 3-year RMST of ODG group was similar to that of LDG group in patients with cT4a (− 1.38 years, *p* = 0.163) or with cT4a and tumor size > 5 cm, whereas the 5-year RMST had significant differences between groups in cT4a patients(− 8.36 years, *P* = 0.005) or cT4a and tumor size > 5 cm patients(4.67 years, *P* = 0.042). In patients with cT4a and tumors > 5 cm, the number of peritoneal recurrences was significantly fewer in the ODG group than in the LDG group (4 vs. 17, *P* = 0.033), and the peritoneal recurrence time and multiple-site recurrence time were both later in the ODG group.

**Conclusion:**

By reducing recurrence, ODG achieves a better survival for GC patients with serous infiltration and tumors larger than 5 cm beyond 3 years after surgery. The present findings can serve as a reference for surgical options and the setting of follow-up time point for clinical studies.

**Supplementary Information:**

The online version contains supplementary material available at 10.1007/s00464-021-08430-0.

Gastric cancer (GC) is the fifth most common cancer worldwide and ranks third among the causes of cancer-related deaths. At present, the influence of time on the efficacy of surgery for GC remains unclear, and the postoperative biological behaviors are also affected by many factors. Although D1 and D2 lymphadenectomy had no difference in survival at the time points of the DUTCH trial, subsequent reports with a prolonged follow-up showed that D2 lymphadenectomy had lower local recurrence and GC-related mortality rates than D1 surgery [1]. The MAGIC trial showed that the survival rate of patients undergoing neoadjuvant chemotherapy and reoperation was comparable to that of patients undergoing direct surgery at about 1 year, but gradually became higher than that in patients undergoing direct surgery beyond 3 years of postoperatively [2]. Similarly, the CLASSIC study showed that the survival curve of patients undergoing XELOX chemotherapy after surgery was stable beyond 3 years, whereas the survival curve of patients who did not receive chemotherapy significantly decreased beyond 3 years [3]. The above randomized clinical trials (RCTs) with long-term follow-up results showed that although patients with GC receiving different treatments showed no difference in oncologic outcomes for a period of time after surgery, extension of the follow-up time revealed that the biological changes caused by different treatments can gradually lead to differences in clinical manifestations, such as death and recurrence patterns. Therefore, studying the oncologic outcomes of GC patients at various time points after surgery can help clinicians in selecting individualized treatment for patients. Radical surgery is currently the main treatment method for GC [4–6]. In the choice of surgical methods, for early distal GC, the use of laparoscopic distal gastrectomy (LDG) has been recognized [7–12], the 5-year oncologic efficacy of which is comparable to that of open distal gastrectomy (ODG) [13]. In advanced distal GC (ADGC), the results of the CLASS-01 trial indicated that the 3-year survival of LDG is comparable to that of ODG [14]. However, in terms of 5-year survival, the 5-year survival rate remains unclear.

Most previous studies used pathologic staging as a stratification factor to study the prognosis of ADGC; however, pathologic staging is not suitable as a selection index for surgical methods because it cannot be obtained before or during surgery, preventing the surgeon from determining the safe margin of the tumor and lymph node dissection before surgery. But the clinical stage and tumor size, which can be evaluated before or during surgery, have been confirmed as prognostic indicators in patients with GC; however, whether they can be used as a basis for selecting surgical methods still needs to be proven in prospective RCTs. Before the results of RCTs are reported, a large-sample retrospective study is performed and propensity score matching (PSM) is used to appropriately adjust for confounding factors to reduce selection bias, which can provide evidence-based information about the long-term oncologic efficacy of surgical methods for patients with ADGC. Therefore, this study aimed to compare the long-term efficacy beyond 3 years of postoperatively of ODG and LDG for ADGC through a multicenter retrospective study, and to explore the clinical stage and tumor size of ADGC patients undergoing different surgical methods. The influence of the prognosis of the nodes provides a reference for the individual selection of surgical methods for patients with ADGC.

## Materials and methods

### General information

We selected two hospitals (Fujian Medical University Union Hospital and Qinghai University Affiliated Hospital) that have facilities for electronic storage of clinical data, including medical records, images, or laboratory data, for all consecutive patients with GC who underwent gastrectomy during the study period. All patients at these institutions who met the inclusion criteria were enrolled. This study was approved by the appropriate Institutional Review Board (Fujian Medical University Union Hospital and Qinghai University Affiliated Hospital). All the patients were well informed and gave their full consent after receiving a verbal explanation of this study and an information document.

**Fig. 1:**
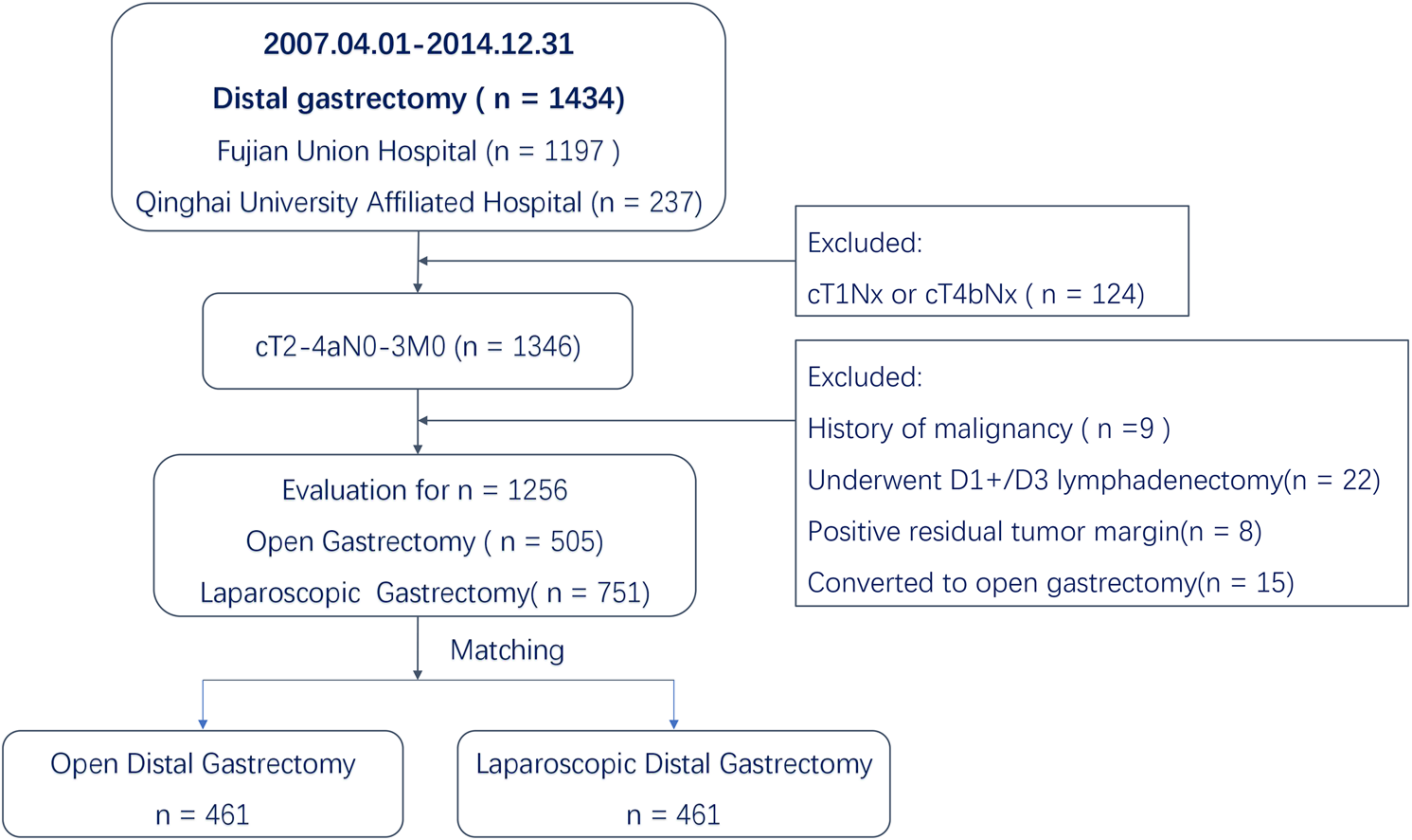
Flow chart of patient inclusion

The patients enrolled in this study had histologically confirmed gastric adenocarcinoma, diagnosed as clinical stage cT2-4aN0-3M0, and had undergone radical distal gastrectomy with D2 lymphadenectomy between April 2007 and December 2014. Eligible patients with the lesion located in the lower or middle third of the stomach on preoperative evaluation, including gastroscopy, full-abdominal CT enhancement and upper gastrointestinal angiography, were included. Conversely, patients with cT1/T4b or unknown stage, positive residual tumor margin, other malignant tumors or a history of malignant tumors, D1 + or D3 lymphadenectomy, and conversion to open surgery were excluded (Fig. 1). Finally, 1256 patients were enrolled, including 505 patients who underwent open surgery and 751 patients who underwent laparoscopic surgery. All the patients selected in this study were performed by surgeons with rich experience in open and laparoscopic radical distal gastrectomy. All patients received preoperative conversation with the surgeon before operation and chose the open or laparoscopic surgical methods and signed the informed consent. In this study, preoperative cT and cN staging of patients was conducted according to the Eighth Union for International Cancer Control tumor-node-metastasis (TNM) classification [15], which was evaluated by two physicians, respectively, in combination with Computerized tomography(CT), Magnetic Resonance Imaging(MRI) and ultrasonic gastroscopy of the patients. If there were differences in the results, PETCT would be used to further confirm the clinical staging if necessary. cT staging was mainly evaluated by ultrasound gastroscopy and CT, and the criteria were: The presence of a mass in the stomach usually is diagnosed as a hypoechoic or dark thickening in one or more layers. A dark thickening extending to but not completely through the fourth layer with a preserved smooth outer echogenic border is associated with a T2 tumor. Complete loss of all the layers, associated with a smooth white outer layer representing the serosal surface, suggests penetration through the muscularis propria into the subserosal fat is consistent with a T3 tumor. If there is extension of the dark wall thickening with loss of the serosal echogenic stripe, the tumor is staged T4a. the criteria of cN + was: tumor involvement if round and/or > 8 mm in short axis diameter in preoperative imaging [16–18]. In this study, the D2 lymph node dissections of LDG and ODG were consistent, and standard D2 lymph node dissections including N0.1–7, 8A, 9, 11p, and 12a were performed in accordance with the Japanese Guidelines for the Treatment of Gastric Cancer.

### Postoperative follow-up

Postoperative follow-up was performed in outpatient and hospital settings, every 3 months within 2 years, every 6 months within 3–5 years, and once a year after 5 years. The vast majority of patients routinely underwent physical examination, laboratory tests, chest radiography, full-abdominal color Doppler ultrasound or full-abdominal CT, and annual gastroscopy. Recurrence was identified by medical history and physical examination in combination with imaging evaluation, cytology, or tissue biopsy (preferred when feasible). The endpoints were overall survival (OS) and disease-free survival (DFS).

### Short-term and long-term efficacy analyses

The incidence of postoperative complications with a severity of grade III or higher according to the Clavien–Dindo classification was assessed [19].

We calculated the life years gained using differences in restricted mean survival time [20] (RMST). The RMST is the mean survival time of all subjects in the study population followed up to *t*, defined as RMST(*t*) = *E*(min(*T, t*)), which is the area under the survival curve of *T* up to time *t*. the RMST(*t*) can be estimated well with the area under the corresponding Kaplan–Meier curve up to time *t*. Note that the area above the survival curve up to time *t* is the restricted mean time lost(RMTL(*t*)), which is *t*-RMST(*t*). With the RMST*(t) *or RMTL*(t)* summary measure, one can then construct the corresponding mean survival time curve over time, which provides a temporal, stochastic profile of T, expressed in units of time, for evaluating, for example, the benefit or safety of a new therapy. The advantage of using such a quantification over the survival rate is the setting of a fixed-time analysis.

We performed landmark analyses to assess outcomes before and beyond 3 years. Landmark analysis was introduced in 1983 by Anderson et al.[21] The goal is to estimate, in an unbiased way, the survival probabilities in ODG and LDG conditional on the group membership of patients at the landmark time (beyond 3 years of postoperatively).

Kaplan–Meier curves were used to analyze the OS and DFS of different surgical methods, and log-rank test was used to test whether there were significant differences between survival curves. Cox regression was used to conduct univariate analysis on the factors affecting OS, and the meaningful results of univariate analysis were incorporated into multivariate analysis to determine the independent prognostic factors that affect OS.

### Propensity score matching (PSM)

PSM [22] was conducted by a biostatistician who was blinded to the outcomes. The propensity score was estimated using a logistic regression model, and a 1:1 (ODG to LDG) matching (matched without replacement) with a caliper of 0.01 standard deviation of the estimated logit was performed. In addition to the propensity score, four preoperative factors (age, body mass index [BMI], clinical *T* and *N* factors) were exactly matched.

### Statistical methods

Continuous variables are expressed as mean ± standard deviation, and categorical variables are expressed as percentages. The chi-square test, Fisher’ s exact probability method, or unpaired *t* test were used to compare the differences in clinicopathologic data between the ODG group and the LDG group. Analyses were performed using SPSS 23.0 and RStudio version 1.1.419 (RStudio Inc.). *P* < 0.05 on both sides was considered to indicate a significant difference.

## Results

### Clinicopathologic data before and after PSM

A total of 1256 patients with ADGC were included in this study. Of them, 505 were categorized into the ODG group and 751 in the LDG group before PSM. Before PSM, the patients in the ODG group had younger age, lower BMI, more advanced stage (including cT stage, cN stage, pT stage), more lymphatic vessel invasion, and higher proportion of those with adjuvant chemotherapy than patients in the LDG group (all *P* < 0.05). After matching, there were 461 patients in each group and the general data between the groups were comparable (*P* > 0.05) (Table 1).

**Table 1:** General information before and after propensity score matching

	Before propensity score matching			After propensity score matching		
	ODG (*n* = 505)	LDG (*n* = 751)	*P*-value	ODG (*n* = 461)	LDG (*n* = 461)	*P*-value
Age			0.008			0.292
< 60 years	282 (55.8)	362 (48.2)		238 (51.6)	222 (48.2)	
≥ 60 years	223 (44.2)	389 (51.8)		223 (48.4)	239 (48.4)	
Gender			0.881			0.885
Female	154 (30.5)	232 (30.9)		324 (70.3)	326 (70.7)	
Male	351 (69.5)	519 (69.1)		137 (29.7)	135 (29.3)	
BMI(Kg/m^2^)^a^	21.0 (± 3.4)	21.9 (± 3.4)	0.001	21.0 (± 3.4)	21.7 (± 3.5)	0.120
Histological type			0.522			0.625
Differentiated	181 (38.9)	303 (40.8)		171 (39.9)	176 (38.3)	
Undifferentiated	284 (61.1)	440 (59.2)		258 (60.1)	284 (61.7)	
Unknow	39	8		32	1	
Size(cm) ^a^	4.3 (± 2.0)	4.1 (± 2.1)	0.080	4.3 (± 2.0)	4.2 (± 2.1)	0.360
cT stage			0.014			0.075
cT2	104 (20.6)	186 (24.8)		104 (22.6)	77 (16.7)	
cT3	128 (25.3)	222 (29.6)		126 (27.3)	130 (28.2)	
cT4a	273 (54.1)	343 (45.7)		231 (50.1)	254 (55.1)	
cN stage			< 0.001			0.838
cN0	174 (34.5)	345 (45.9)		170 (36.9)	173 (37.5)	
cN +	331 (65.5)	406 (54.1)		291 (61.1)	288 (62.5)	
pT stage			< 0.001			0.762
pT1	120 (23.8)	226 (30.1)		120 (26.0)	124 (26.9)	
pT2-3	213 (42.1)	346 (47.4)		213 (46.2)	202 (43.8)	
pT4	172 (34.1)	169 (22.5)		128 (27.8)	135 (29.3)	
pN stage			0.429			0.377
pN0	165 (32.4)	270 (36.0)		162 (35.1)	161 (34.9)	
pN1	107 (21.2)	131 (17.4)		93 (20.2)	74 (16.1)	
pN2	100 (19.8)	149 (19.8)		88 (19.1)	93 (20.2)	
pN3	133 (26.3)	201 (26.8)		118 (25.6)	133 (28.9)	
Lymphovascular invasion			0.005			0.091
Negative	353 (68.1)	579 (77.1)		338 (73.3)	360 (78.1)	
Positive	152 (31.9)	173 (22.9)		123 (28.5)	101 (20.1)	
Preoperational ALB,g/L^a^	38.9 (± 5.1)	39.4 (± 5.6)	0.161	38.9 (± 5.3)	38.7 (± 5.5)	0.552
Preoperational Hb, g/L			0.025			0.188
< 120	113 (22.3)	231 (30.8)		91 (19.7)	109 (23.6)	
≥ 120	262 (51.9)	392 (52.2)		245 (53.1)	235 (51.0)	
CEA (U/mL)			0.810			0.848
< 5.0	193 (82.8)	512 (83.5)		190 (82.6)	273 (82.0)	
≥ 5.0	40 (17.2)	101 (16.5)		40 (17.4)	60 (18.0)	
Unknown	272	138		231	128	
Chemotherapy			0.005			0.225
No	165 (32.7)	190 (25.3)		150 (32.5)	133 (28.9)	
Yes	340 (67.3)	561 (74.7)		311 (67.5)	328 (71.1)	

Values in parentheses are percentages unless indicated otherwise

*BMI* body mass index, *cT* clinical T staging, *cN* clinical N staging, *pT* pathological T staging, *pN* pathological N staging, *ALB* albumin, *Hb* hemoglobin, *CEA* Carcinoma Embryonic Antigen

^a^Values are standard deviation

### Short-term outcome of patients after matching

Supplemental Table 1 showed that the ODG group had a longer operation time (199.0 vs. 169.8 min, *P* < 0.001) and more bleeding volume (121.3 vs. 78.7 mL, *P* < 0.001) than the LDG group. Moreover, the fluid intake time (5.3 vs. 4.7 days, *P* < 0.001), drainage tube removal time (8.7 vs. 8.3 days, *P* = 0.001), hospital stay (14.1 vs. 13.0 days, *P* = 0.027), and postoperative recovery were longer in the ODG group. The number of lymph node dissections and the postoperative exhaust time were comparable in the two groups. A total of 80 cases of postoperative complications occurred in the ODG group, including 12 cases of Clavien–Dindo grade III–IV. Meanwhile, 68 cases of postoperative complications occurred in the LDG group, 9 of which were grade III–IV, the rate of complications were comparable in the two groups (*P* = 0.535).

### Survival comparison between the two groups after matching

The median follow-up time for the entire group of patients was 88 months (0–137 months).The Kaplan–Meier survival curve showed that the 3- and 5-year OS and DFS in the ODG group were comparable to those in the LDG group (3-year OS: 74.2% vs. 76.1%, *P* = 0.259; 3-year DFS: 63.2% vs. 65.1%, *P* = 0.331; 5-year OS: 64.8% vs. 67.6%, *P* = 0.369; 5-year DFS: 62.8% vs. 63.6%, *P* = 0.469) (Supplemental Fig. 1).

**Fig. 2:**
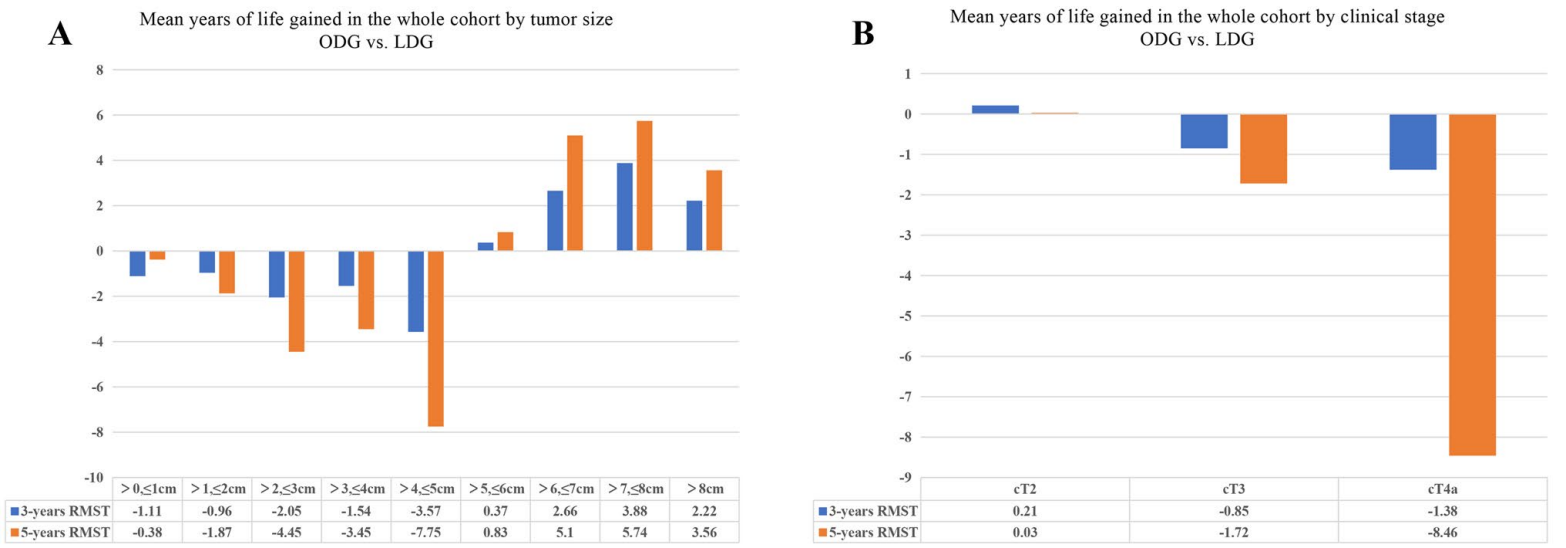
The restricted mean survival time in whole cohort

Figure 2A shows the results of RMST analysis on the effect of tumor size on the survival of patients with different surgical methods. At 1 cm intervals, when the tumor size was ≤ 5 cm, the 3 -and 5-year RMST of the ODG group were shorter than those of the LDG group (3-year RMST: − 1.11, − 0.96, − 2.05, − 1.54, and − 3.57 years; 5-year RMST: − 0.38, − 1.87, − 4.45, − 3.45, and − 16.75 years). When the tumor size was > 5 cm, the 3 and 5-year RMST of the ODG group were longer than those of the LDG group (3-year RMST: 0.37, 2.66, 3.88, and 2.22 years; 5-year RMST: 0.83, 5.10, 5.74, and 3.56 years). Therefore, we analyzed the survival with a tumor size of 5 cm as the cutoff point.

Figure 2B shows the results of stratified analysis according to cT stage. The 3-year RMST of cT2-4a patients in the ODG and LDG groups were comparable (RMST: cT2, 0.21 year, *P* = 0.835; cT3, − 0.850 year, *P* = 0.233; cT4a, − 0.138 year, *P* = 0.16). In the 5-year RMST, although the two groups were comparable in cT2 and cT3 (RMST: cT2, 0.03 year, *P* = 0.987; cT3, − 1.72 years, *P* = 0.368), the RMST in cT4a in the ODG group within 5 years was statistically different from that in the LDG group (RMST: cT4a, − 8.46 years, *P* = 0.005).

Forest chart shows there was no difference in 3-year OS between ODG and LDG in patients with cT4a or tumor > 5 cm, but there was a statistical difference in 5-year OS between the two groups (*P* < 0.05) (Supplemental Fig. 2).

### Subgroup analysis of cT4a patients after matching

Supplemental Table 2 showed that the operation time of the ODG group was significantly longer than that of the LDG group (200.1 vs. 180.9 min, *P* = 0.048) in cT4a patients. In terms of postoperative complications, 33 cases occurred in both the ODG and LDG groups, of which three cases were Clavien–Dindo grade III–IV, with no statistically significant difference (*P* = 0.982).

**Fig. 3:**
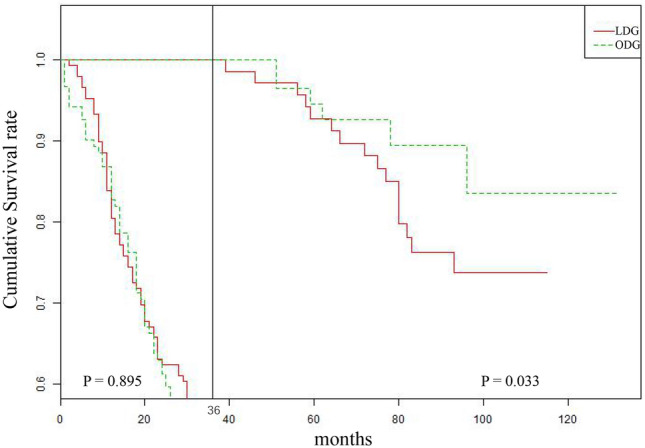
Landmark analysis in patients with cT4a/tumor size > 5 cm

In patients with cT4a, when the tumor was > 5 cm, there was no difference in the 3-year OS and DFS between the two groups (3-year OS: 53.4% vs. 37.9%, *P* = 0.216; 3-year DFS: 55.2% vs. 45.1%, *P *= 0.431); however, the 5-year OS and DFS of the ODG group were significantly better than those of the LDG group (5-year OS: 49.8% vs. 37.1%, *P* = 0.033; 5-year DFS: 52.2% vs. 38.2%, *P* = 0.045) (Supplemental Fig. 3). Using RMST to verify this result. In patients with cT2-3 patients, the RMST of ODG was all comparable to LDG (P > 0.05) (Supplemental Fig. 4A). In patients with cT4a and tumors ≤ 5 cm, the 3-year RMST and 5-year RMST in ODG group were all shorter than LDG group(3-year RMST:− 2.11, *P* = 0.265, 5-year RMST: − 6.42, *P* = 0.012). In patients with cT4a and tumors > 5 cm, the 3-year RMST in the ODG group was comparable to that in the LDG group (3-year RMST: 0.98 year, *P* = 0.480), but the 5-year RMST in the ODG group was significantly longer than that in the LDG group (5-year RMST: 4.67 years, *P* = 0.042) (Supplemental Fig. 4B). Figure 3 shows the results of further landmark analysis. In patients with cT4a and tumors > 5 cm, the 3-year OS of patients in the ODG and LDG groups was comparable (*P* = 0.895), and the OS beyond 3 years of postoperatively was significantly better in the ODG group than in the LDG group (*P* = 0.033).

### Analysis of survival risk factors in cT4a patients after matching

Supplemental Table 3 shows the results of multifactor risk analysis performed to study the impact of ODG and LDG on the OS of cT4a patients, including the factors (age, BMI, tissue type, Tumor size, cN, operation time, intraoperative blood loss, pT, pN, surgery, prealbumin, preoperative hemoglobin, CEA, and postoperative complications), that affect survival in these patients. The results showed that ODG was a risk factor for the 3 and 5 year OS in cT4a patients (3-year OS, hazard ratio [HR] 1.67 [0.65, 4.26]; 5-year OS, HR 1.13 [0.58, 2.50]).

Further analysis of the factors influencing OS in patients with cT4a and tumor size > 5 cm, including all the factor in Model 1 except tumor size, was performed (*P* < 0.05). The results showed that ODG was a protective factor of 5-year OS in patients with cT4a and tumor size > 5 cm (5-year OS, HR 0.08 [0.01, 0.54]).

### Recurrence after matching

**Fig. 4:**
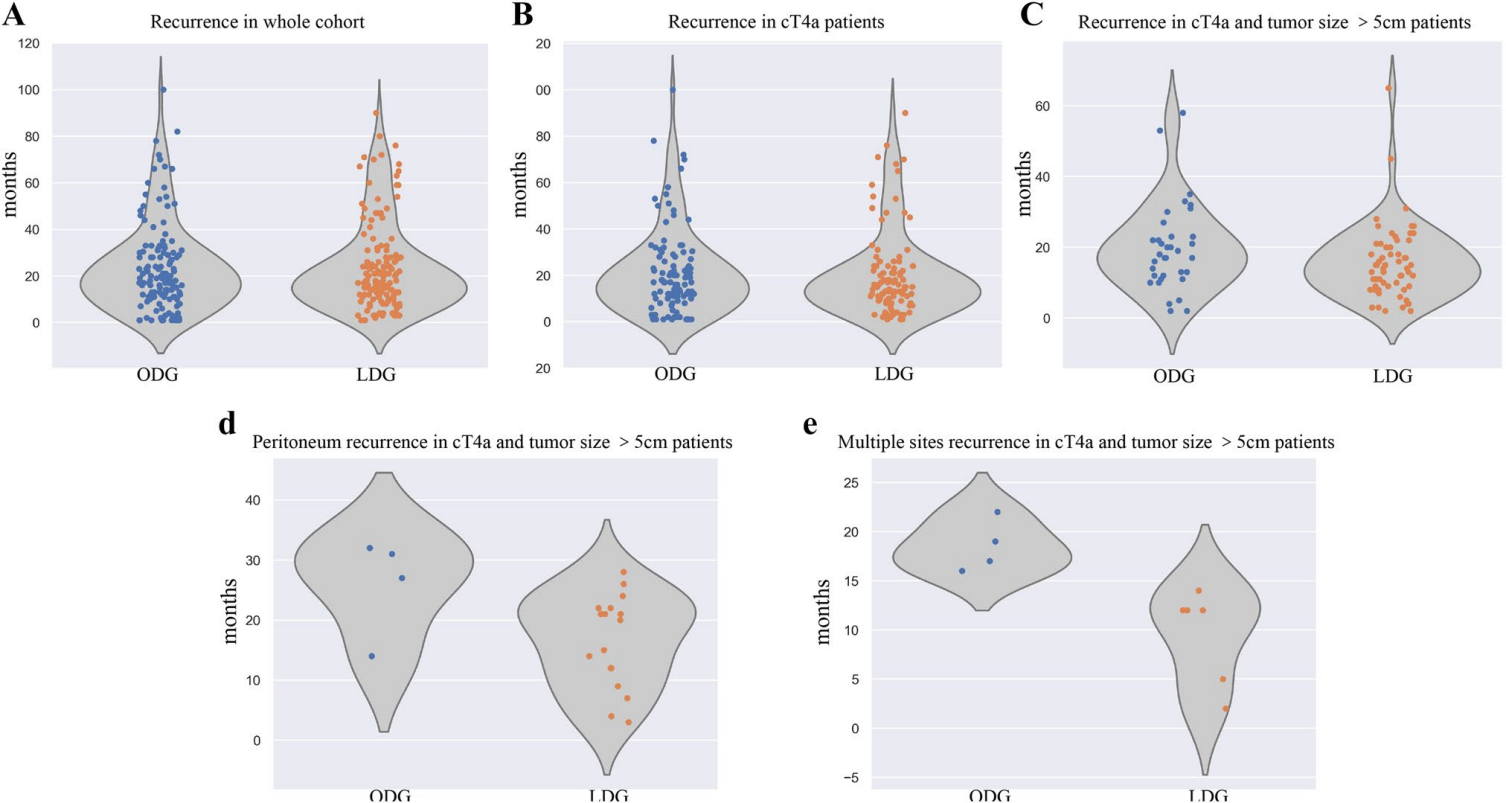
Violin plot of recurrence

Analysis of recurrence after matching showed that patients in the ODG and LDG group had similar in overall recurrence (137 vs. 140, *P* = 0.286), local recurrence (44 vs. 33, *P* = 0.234), peritoneal recurrence (32 vs. 46, *P* = 0.123), multiple-site recurrence (13 vs. 11, *P *= 0.837), and other or unknown-site recurrence (48 vs. 50, *P* = 0.915) (Supplemental Table 4). The overall recurrence time distribution was similar in the two groups (Fig. 4A). The same results showed that in cT4a patients, the two groups showed the similar number of recurrences and similar time of recurrence at each site (Supplemental Table 5, Fig. 4B).

cT4a patients were further stratified. Among patients with cT4a and tumors > 5 cm, the ODG and LDG groups showed non-significant differences in total recurrence (35 vs. 60, *P* = 0.178, Fig. 4C), local recurrence (13 vs. 15, *P* = 0.682), and other or unknown-site recurrence (14 vs. 22, *P* = 0.711). In terms of peritoneal recurrence, the number of recurrences in the ODG group was significantly fewer than that in the LDG group (4 vs. 17, *P* = 0.033) (Supplemental Table 6), and the recurrence time was later than in the LDG group (Fig. 4D).With respect to multiple-site recurrences, the multiple-site recurrence time was later in the ODG group (Fig. 4E). Supplemental Fig. 5 compares the recurrence risk of patients in the ODG and LDG groups. In cT4a patients, the recurrence risk was comparable between the ODG and LDG groups; however, in cT4a patients with tumors > 5 cm, the recurrence risk of the ODG group was significantly lower than that of the LDG group. A further landmark analysis was performed to compare the OS of patients with cT4a and tumors > 5 cm, excluding the patients who suffered peritoneal recurrence and multiple-site recurrence, there was not significantly different within 3 years and beyond 3 years of postoperatively in OS between two groups (*P* = 0.715 and *P* = 0.500, respectively) (Supplemental Fig. 6).

## Discussion

As surgery is the main treatment method for GC, how it affects the survival of GC patients at various time points needs to be confirmed by research. At present, there have been reports on the completion of 3-year follow-up data in two multicenter, large-sample RCTs in China and South Korea. Class 01 reported that the 3-year OS of ODG and LDG were 85.2% and 83.1%, respectively, while the 3-year DFS were 77.8% and 76.5%, both comparable [14]. KLASS-02 also reported similar 3-year OS with LDG and ODG, 90.6% and 90.3%, respectively [23], while Japanese JLSSG0901 is still in the process of case enrollment. Thereby, exploring the most appropriate surgical option for GC patients is the focus of research. In the past 20 years, laparoscopy has become a recognized surgical method for the treatment of early lower GC [7–12]. However, the suitable surgical method for ADGC is still controversial [24]. Our analysis of large-sample data from two centers in China through strict PSM showed that the 3-year OS and DFS of patients with ADGC were comparable in the ODG and LDG groups, which is similar to the results of the CLASS-01 and KLASS-02 trial. However, in terms of the 5-year oncologic efficacy, large-scale RCTs are still lacking. Therefore, this study further investigated the 5-year difference in oncologic efficacy between LDG and ODG.

The clinical stage and tumor size can be more easily measured before or during surgery. Existing studies have shown that these are prognostic indicators in patients with GC [25–32]. Meantime, clinical stage and tumor size can also significantly affect the surgery process. Studies have shown that tumors with late stages and large diameters may hinder the operation of laparoscopic linear devices and interfere with the surgeon’s visual field, and could also increase the difficulty of using laparoscopic instruments for grasping the thickened stomach wall [33]. However, whether these effects will influence the prognosis of GC patients on different surgery has not been confirmed by studies. The results of the study showed that when the patient’s clinical stage was T4a and the tumor was > 5 cm, although the 3-year OS and DFS were comparable between the two groups, ODG was significantly better than LDG in terms of the 5-year OS and DFS. Landmark analysis also showed that ODG was significantly better than LDG with respect to OS beyond 3 years of postoperatively. We further used RMST to quantify this advantage, and the results showed that compared with LDG, ODG can provide patients with an additional 4.67 months of survival time within 5 years. The results of the above studies suggest that clinicians choose ODG to provide better survival beyond 3 years of postoperatively for ADGC patients with an advanced preoperative stage and a large tumor. This observation not only provides clinicians with an extremely important basis for surgical decision-making, but also provides researches in the field of GC with a principle for clinical research. That is, if the follow-up time of clinical research is only ≤ 3 years, the results may accurately reveal the true prognosis of GC. Therefore, it is recommended that when formulating a prospective research plan or conducting a retrospective study, researchers should do follow-up for the patients for at least 3 years to avoid one-sided research results caused by the changes in tumor biological characteristics over time after surgery, or to avoid inaccurate or even diametrically opposite results.

Thereby, determining the clinical stage and tumor size before surgery is particularly important. Previous studies have shown that endoscopic ultrasonography (EUS) has a unique advantage in distinguishing T1-2 and T3-4 stage GC, with an overall sensitivity of 86% and specificity of 91% [34, 35]. However, Han et al.[36] showed that EUS may overestimate or underestimate the staging [37]. Therefore, at present, most researchers believe that EUS combined with CT can improve the accuracy preoperative T staging [38–40]. Previous research reported that the accuracy of preoperative cT staging and postoperative pT staging was 41.0–86.84%, [35, 41, 42], and the accuracy of lymph node metastasis was 70–75% [40, 43, 44]. The accuracy of cT staging and cN staging in this study were 72.7% (670/922) and 78.2% (721/922), which were similar to those researches. Conversely, Hu [45] believed that direct visual inspection during laparoscopic exploration is the most accurate way to judge the preoperative stage. Therefore, when imaging determined that the GC stage is cT4a and the tumor is > 5 cm, laparoscopic surgery should be carefully selected. However, when there are difficulties in preoperative imaging judgment, such as color-diffused-type GC with serosal defect characteristics, [46] it may be difficult to determine the depth of tumor infiltration. In this case, laparoscopic exploration should be performed first during surgery. When the tumor is found to invade the serosa, and the diameter of the tumor is > 5 cm, conversion to open surgery should be performed in a timely manner.

We further studied the possible reasons why ODG prolongs the survival of patients with cT4a and tumors > 5 cm compared with LDG beyond 3 years of postoperatively. The analysis found that although the overall recurrence rates of ODG and LDG were similar, the number of peritoneal recurrences in the ODG group was significantly fewer than that in the LDG group. At the same time, peritoneal recurrence and multiple-site recurrence in the ODG group occurred at around 30 and 17 months, respectively, which were significantly longer than the 21 and 12 months in the LDG group, respectively. We further performed a landmark analysis to compare the OS of the two groups after excluding patients with peritoneal recurrence and multiple-site recurrence. The results showed that the ODG and LDG groups had comparable OS within 3 years and beyond 3 years of postoperatively. Therefore, we believe that the reason for the decreased survival rate of patients with late-stage and large-sized tumors in the LDG group may be the increased risk of peritoneal recurrence and multiple-site recurrence due to laparoscopic surgery. For these patients (cT4a and tumor size > 5 cm), open surgery is a more appropriate choice. Sasako's point of view supports our results. [33] He believes that advanced GC is poorly differentiated and prone to intra-abdominal spread and extranodal metastasis. Careful dissection of lymph nodes overlapped by a complete membranous structure requires high levels of laparoscopic skill. At the same time, when dealing with cases of large tumors or large metastatic nodules, contacting the primary tumor with a metal gripper may lead to an increased risk of cancer cell overflow, leading to an increased likelihood of peritoneal metastasis. Therefore, we recommend ODG for patients with a clinical stage of cT4a and a tumor size of > 5 cm in order to reduce the rate of peritoneal recurrence and multiple-site recurrence, thereby improving survival.

Although this study used multicenter, large-sample data and adopted strict PSM for the analysis, several limitations remain. First, even after a very strict matching of the underlying factors, it is still not guaranteed that all confounding factors have been adjusted for in our analysis, such as the selection of open or laparoscopy is subject to the inevitable bias of retrospective studies. To evaluate the accuracy of the study, it is necessary to perform an RCT for further verification. Second, this study was based on a database of Chinese hospitals and skilled surgeons. The number of GC surgeries significantly differs between the East and the West, and the obesity rate in Western countries is also high. Therefore, the applicability of the present study results to all institutions or other regions worldwide needs to be further evaluated. However, we still believe that the conclusions obtained in this study have great clinical significance. We recommend ODG treatment for GC patients with a preoperative clinical stage of cT4a and a tumor size > 5 cm.

## Supplementary Information

Below is the link to the electronic supplementary material.

Supplementary file1 (TIF 958 kb). Supplemental figure1. Overall survival and disease-free survival in whole cohort

Supplementary file2 (TIF 5263 kb). Supplemental figure2. Forest chart of 3-year and 5-year in whole cohort

Supplementary file3 (TIF 1049 kb). Supplemental figure3. Overall survival and disease-free survival in cT4a patients

Supplementary file4 (TIF 164 kb). Supplemental figure4. Mean year of life gained in cT4a patients stratify according to tumor size

Supplementary file5 (TIF 1251 kb). Supplemental figure5. Recurrence cumulative risk in cT4a patients and stratify according to tumor size

Supplementary file6 (TIF 593 kb). Supplemental figure6. Landmark analysis of patients with cT4a and tumors >5 cm, excluding the peritoneal recurrence and multiple-site recurrence patients

Supplementary file7 (DOC 20 kb)

Supplementary file8 (DOC 49 kb)

Supplementary file9 (DOC 17 kb)

Supplementary file10 (DOC 14 kb)

Supplementary file11 (DOC 14 kb)

Supplementary file12 (DOC 14 kb)

## Abbreviations

*ADGC*: Advanced distal gastric cancer
*LDG*: Laparoscopic distal gastrectomy
