# Supplementary figures and images for: Potential survival benefits of open over laparoscopic radical gastrectomy for gastric cancer patients beyond three years after surgery: result from multicenter in-depth analysis based on propensity matching
Source: Surg Endosc. 2021 Jun 3;36(2):1456–65. doi: 10.1007/s00464-021-08430-0 (PMC8758649; doi:10.1007/s00464-021-08430-0)

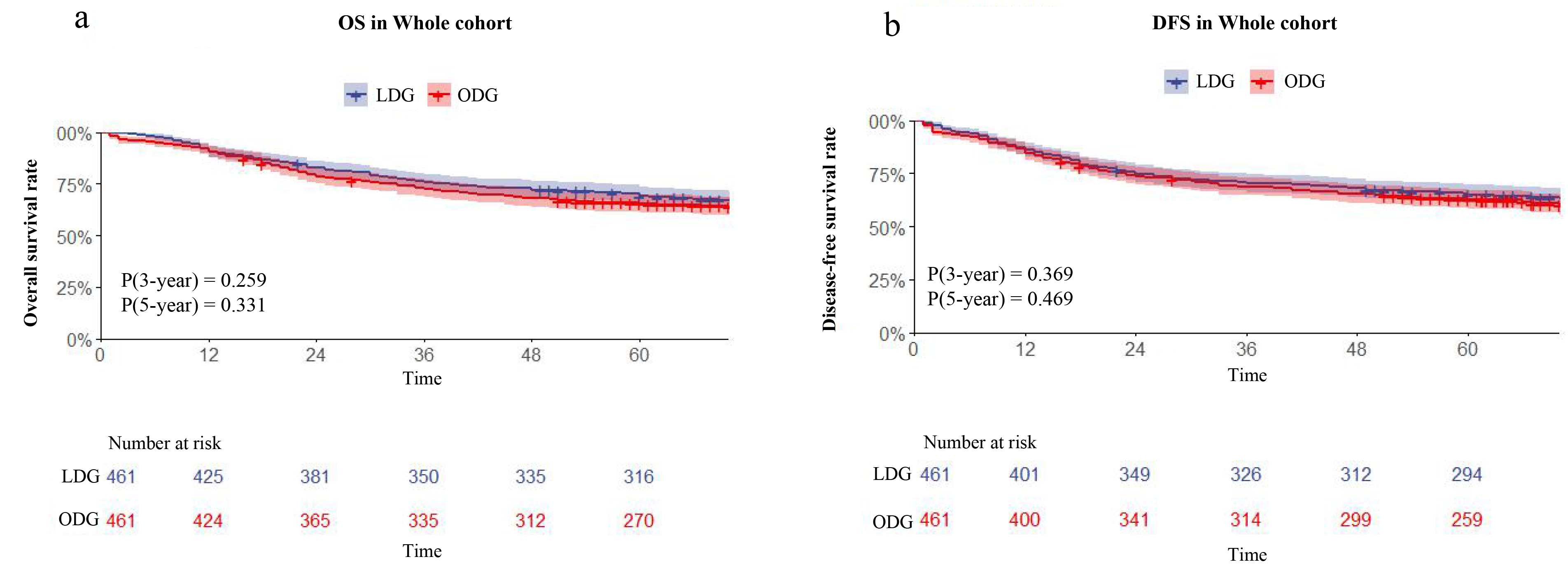

Supplement: Supplementary file 1 — Supplementary file1 (TIF 958 kb). Supplemental figure1. Overall survival and disease-free survival in whole cohort [file 464_2021_8430_MOESM1_ESM.tif]

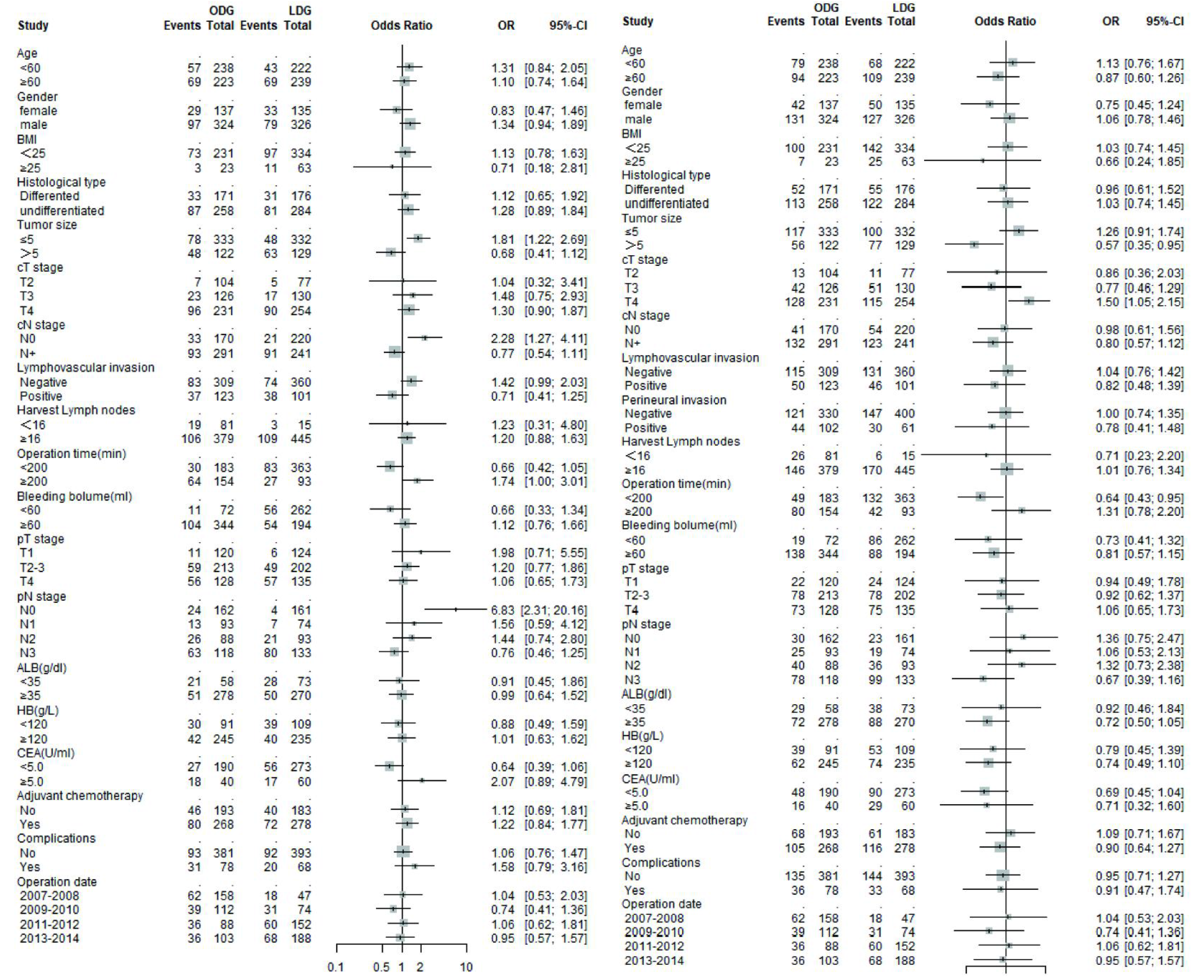

Supplement: Supplementary file 2 — Supplementary file2 (TIF 5263 kb). Supplemental figure2. Forest chart of 3-year and 5-year in whole cohort [file 464_2021_8430_MOESM2_ESM.tif]

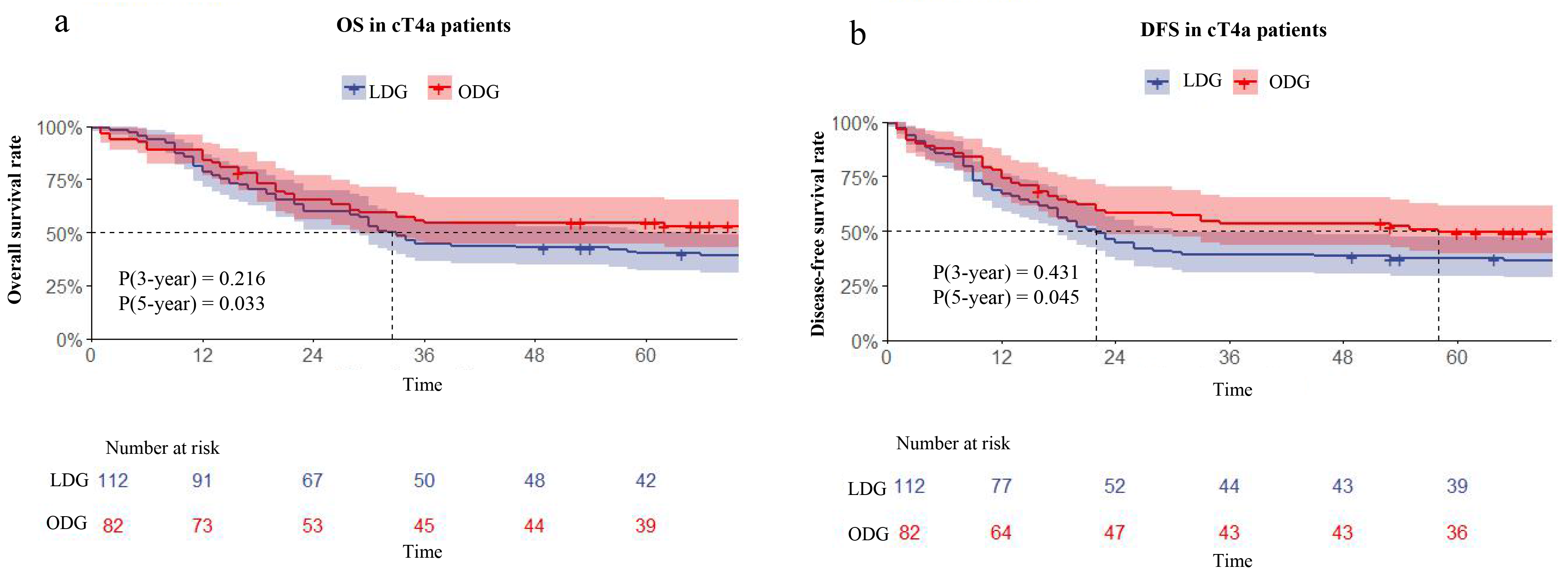

Supplement: Supplementary file 3 — Supplementary file3 (TIF 1049 kb). Supplemental figure3. Overall survival and disease-free survival in cT4a patients [file 464_2021_8430_MOESM3_ESM.tif]

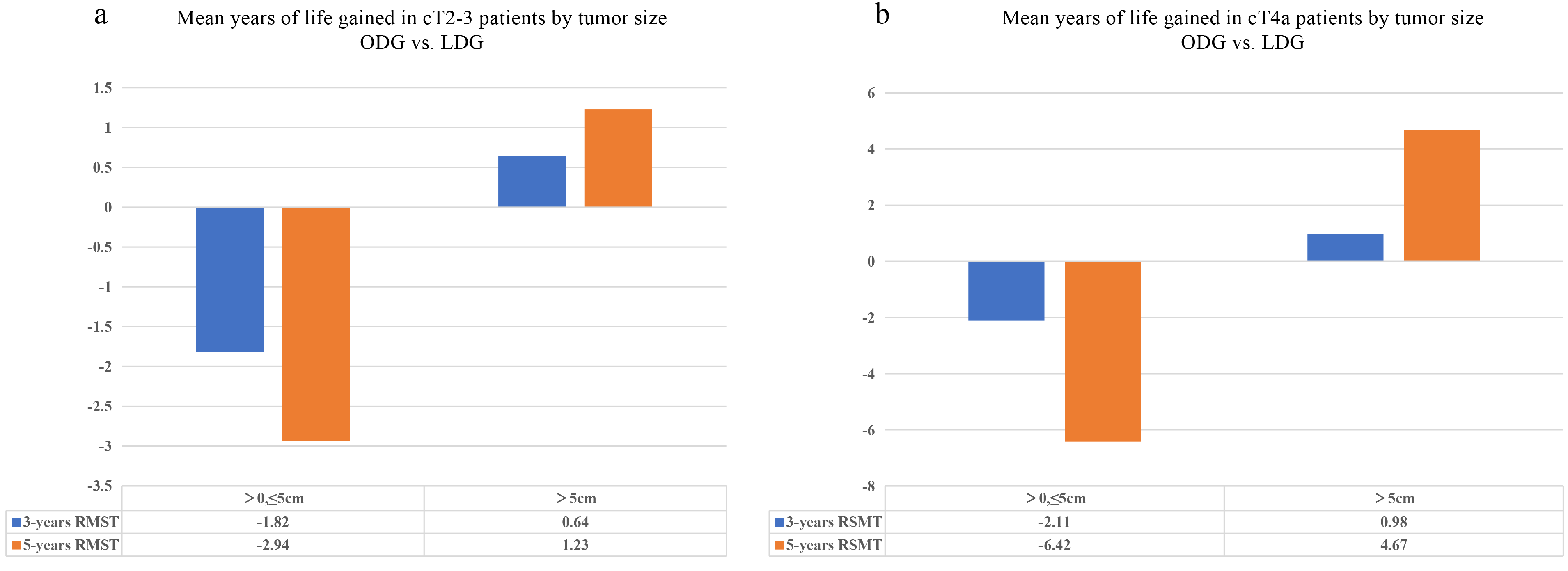

Supplement: Supplementary file 4 — Supplementary file4 (TIF 164 kb). Supplemental figure4. Mean year of life gained in cT4a patients stratify according to tumor size [file 464_2021_8430_MOESM4_ESM.tif]

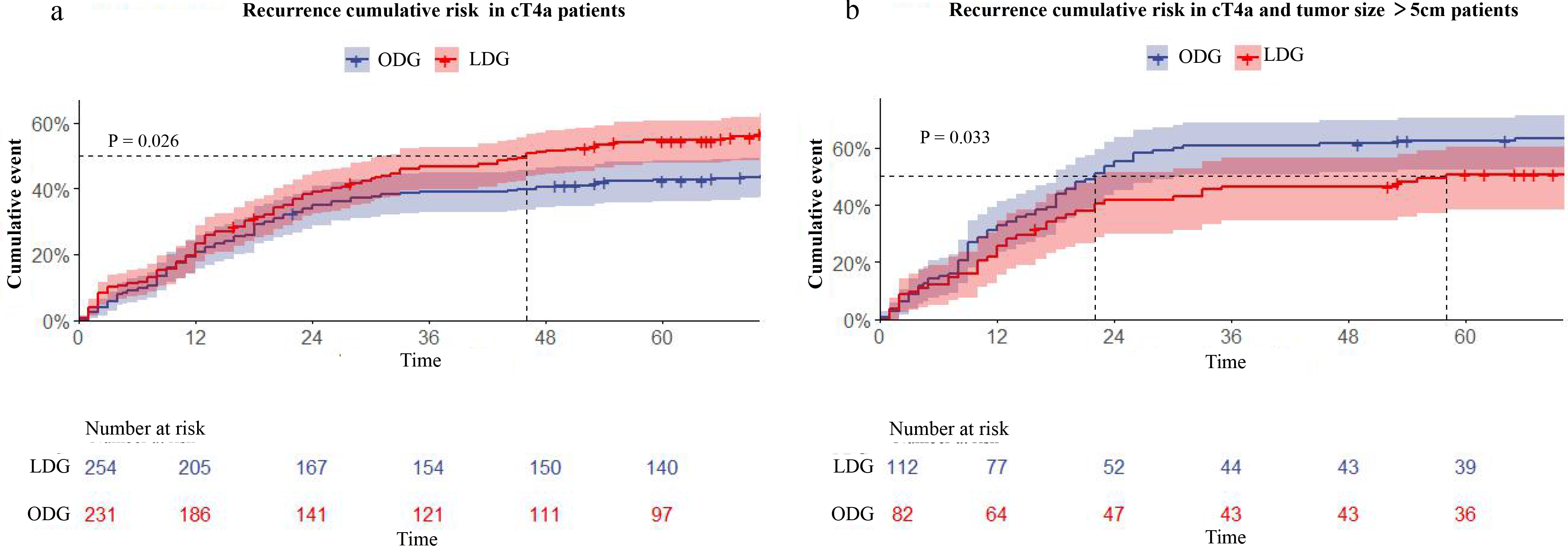

Supplement: Supplementary file 5 — Supplementary file5 (TIF 1251 kb). Supplemental figure5. Recurrence cumulative risk in cT4a patients and stratify according to tumor size [file 464_2021_8430_MOESM5_ESM.tif]

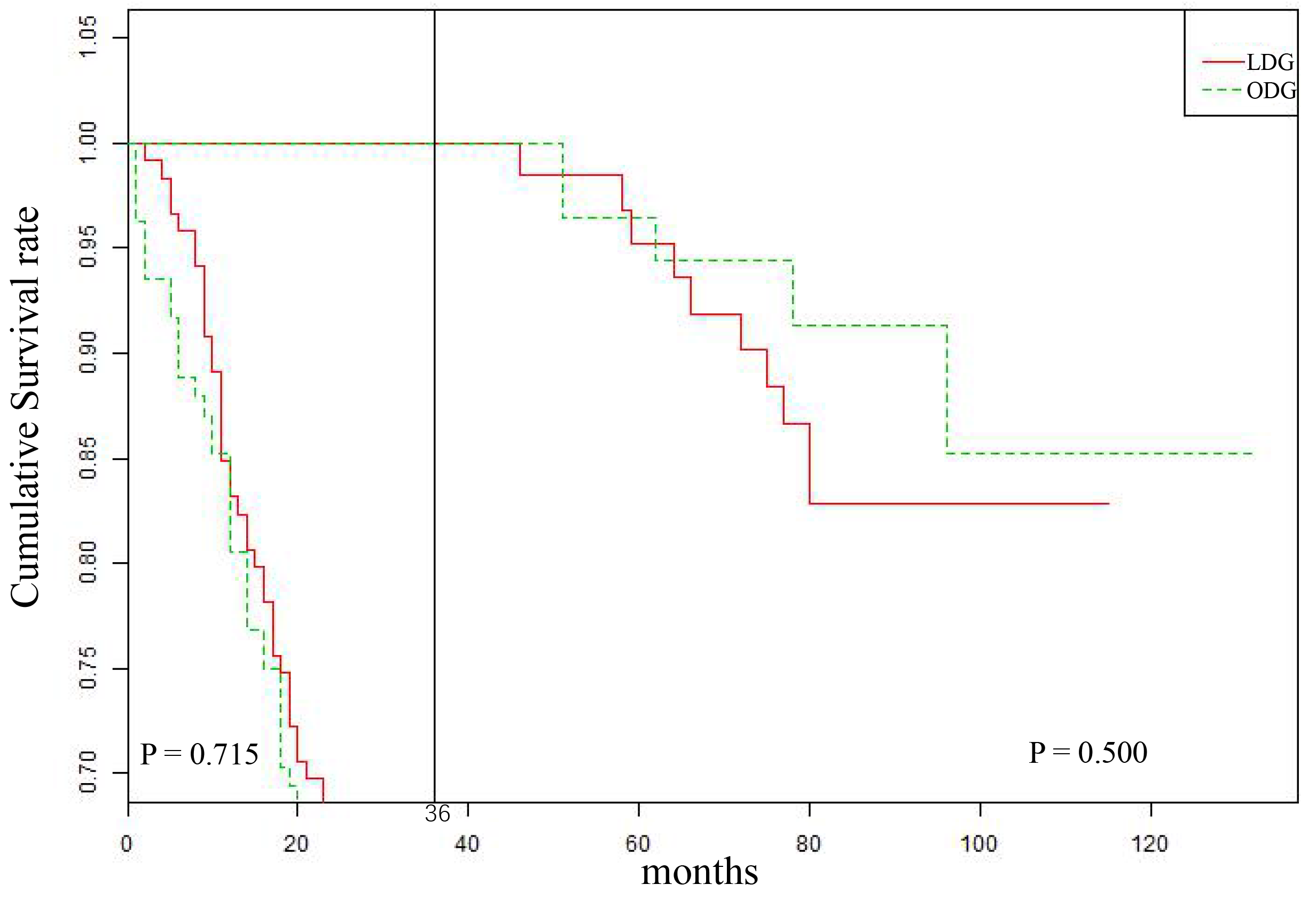

Supplement: Supplementary file 6 — Supplementary file6 (TIF 593 kb). Supplemental figure6. Landmark analysis of patients with cT4a and tumors >5 cm, excluding the peritoneal recurrence and multiple-site recurrence patients [file 464_2021_8430_MOESM6_ESM.tif]
