# Supplementary material for: Potential survival benefits of open over laparoscopic radical gastrectomy for gastric cancer patients beyond three years after surgery: result from multicenter in-depth analysis based on propensity matching
Source: Surg Endosc. 2021 Jun 3;36(2):1456–65. doi: 10.1007/s00464-021-08430-0 (PMC8758649; doi:10.1007/s00464-021-08430-0)
Supplement: Supplementary file 7 — Supplementary file7 (DOC 20 kb) [file 464_2021_8430_MOESM7_ESM.doc]

**Supplemental table1.** Operation and Postoperative recovery after propensity score matching

|  | ODG (n=461) | LDG (n=461) | P-value |
| --- | --- | --- | --- |
| Operation time, min* | 176.0(±62.75) | 169.8(±47.1) | 0.090 |
| Blood loss, ml * | 121.3(±115.5) | 78.7(±98.9) | <0.001 |
| Harvest Lymph nodes* | 30.5(±12.1) | 31.3(±10.8) | 0.348 |
| First Exhaust Time, d* | 4.0(±1.4) | 3.8(±1.2) | 0.054 |
| Fluid diet, d* | 5.3(±1.9) | 4.7(±1.5) | <0.001 |
| Drainage Tube Removal Time, d* | 8.7(±2.4) | 8.3(±1.7) | 0.001 |
| Hospital Stay, d* | 14.1±8.6 | 13.0±8.3 | 0.027 |
| Complications |  |  | 0.535 |
| No | 381(82.6) | 393(85.2) |  |
| Clavien-Dindo grade II-III | 68(14.8) | 59(12.8) |  |
| Clavien-Dindo grade III-IV | 12(2.6) | 9(2.0) |  |

Values in parentheses are percentages unless indicated otherwise; *values are standard deviation.
