# Supplementary material for: Potential survival benefits of open over laparoscopic radical gastrectomy for gastric cancer patients beyond three years after surgery: result from multicenter in-depth analysis based on propensity matching
Source: Surg Endosc. 2021 Jun 3;36(2):1456–65. doi: 10.1007/s00464-021-08430-0 (PMC8758649; doi:10.1007/s00464-021-08430-0)
Supplement: Supplementary file 8 — Supplementary file8 (DOC 49 kb) [file 464_2021_8430_MOESM8_ESM.doc]

**Supplemental table 2.** General information in cT4a patients after propensity score matching

|  | ODG (n=171) | LDG (n=164) | P-value |
| --- | --- | --- | --- |
| **Age(y)** |  |  | 0.620 |
| ＜60years | 84(49.1) | 85(51.8) |  |
| ≥60years | 87(50.9) | 79(48.2) |  |
| **Gende**r |  |  | 0.731 |
| female | 53(31.0) | 48(29.3) |  |
| male | 118(69.0) | 116(70.7) |  |
| **BMI(kg/m^2^)** | 21.5(±2.7) | 22.3(±3.2) | 0.074 |
| **Histological type** |  |  | 0.372 |
| Differentiated | 46(26.9) | 55(33.5) |  |
| Undifferentiated | 113(66.1) | 109(66.5) |  |
| Unknow | 12(7.0) | 0(0) |  |
| **Size(mm)** | 5.1(±1.9) | 5.0(±2.2) | 0.864 |
| **cN stage** |  |  | 0.129 |
| cN0 | 47(27.5) | 48(29.3) |  |
| cN+ | 124(72.5) | 116(70.7) |  |
| **Lymphovascular invasion** |  |  | 0.235 |
| Negative | 128(74.9) | 123(75.0) |  |
| Positive | 31(18.1) | 41(2150) |  |
| Unknow | 12(7.0) | 0(0.0) |  |
| **Harvest Lymph nodes** | 29.0(±11.0) | 30.6(±9.9) | 0.474 |
| **pT stage** |  |  | 0.085 |
| pT1 | 3（1.8） | 10（6.1） |  |
| pT2-3 | 59（36.0） | 66（40.3） |  |
| pT4 | 109（66.5） | 88（53.7） |  |
| **pN stage** |  |  | 0.097 |
| pN0 | 38(23.2) | 20(11.7) |  |
| pN1 | 30(18.3) | 37(21.6) |  |
| pN2 | 37(22.6) | 39(22.8) |  |
| pN3 | 59(36.0) | 47(27.5) |  |
| **Preoperational ALB(g/mL)** | 37.3(±5.2) | 37.1(±7.4) | 0.081 |
| **Preoperational Hb(g/L)** |  |  | 0.589 |
| **＜120** | 78(45.6) | 70(42.7) |  |
| **≥120** | 93(54.4) | 94(57.3) |  |
| **CEA(U/mL)** |  |  | 0.102 |
| ＜5.0 | 42(24.5) | 79(48.2) |  |
| ≥5.0 | 21(12.3) | 22(13.4) |  |
| unknow | 108(63.2) | 63(38.4) |  |
| **Chemotherapy** |  |  | 0.179 |
| No | 40(23.4) | 49(29.9) |  |
| Yes | 131(76.6) | 115(70.1) |  |
| **Operation time** | 200.1(±59.01) | 180.9(±65.1) | 0.048 |
| **Blood loss(mL)** | 107.3(±114.4) | 97.7(±70.8) | 0.481 |
| **First Exhaust Time** | 3.7(±1.4) | 3.8(±1.4) | 0.674 |
| **Fluid diet** | 5.3(±1.5) | 5.0(±2.0) | 0.234 |
| **Drainage Tube Removal Time** | 8.9(±2.3) | 8.4(±3.0) | 0.206 |
| **Hospital Stay** | 14.8±8.0 | 13.7±8.1 | 0.330 |
| **Complications** |  |  |  |
| No | 138(80.7) | 131(79.9) | 0.982 |
| Clavien-Dindo grade II-III | 30(17.5) | 30(18.3) |  |
| Clavien-Dindo grade III-IV | 3(1.8) | 3(1.8) |  |

Values in parentheses are percentages unless indicated otherwise; *values are standard deviation. Abbreviation: BMI, body mass index, cT: clinical T staging, cN: clinical N staging, pT: pathological T staging, pN: pathological N staging, ALB: albumin, Hb: hemoglobin, CEA: Carcinoma Embryonic Antigen.
