# Supplementary material for: Potential survival benefits of open over laparoscopic radical gastrectomy for gastric cancer patients beyond three years after surgery: result from multicenter in-depth analysis based on propensity matching
Source: Surg Endosc. 2021 Jun 3;36(2):1456–65. doi: 10.1007/s00464-021-08430-0 (PMC8758649; doi:10.1007/s00464-021-08430-0)
Supplement: Supplementary file 9 — Supplementary file9 (DOC 17 kb) [file 464_2021_8430_MOESM9_ESM.doc]

**Supplemental table 3.** The influence of ODG or LDG on 3-years and 5-years OS in different populations

| Surgery | Model 1 in cT4a patients | |  | Model 2 in cT4a and Tumor size＞5cm patients | |
| --- | --- | --- | --- | --- | --- |
|  | 3 years HR(95%CI) | 5 years HR(95%CI) |  | 3 years HR(95%CI) | 5 years HR(95%CI) |
| ODG | 1.67(0.65,4.26) | 1.13(0.58,2.50) |  | 0.60(0.24,1.53) | 0.08(0.01,0.54) |
| LDG | 1.00(reference) | 1.00(reference) |  | 1.00(reference) | 1.00(reference) |

Adjusted hazard ratio, and 95% confidence interval.

**Model** 1.In cT4a patients, adjustment was made for age, gender, body mass index, histological type cN, lymphovascular invasion, operation time, blood loss, pT, pN, tumor size ,ALB, Hb, CEA, Chemotherapy according to univariable analysis

**Model 2**.In cT4a and Tumor size＞5cm patients, include all the index in Model 1 except tumor size.
