# Supplementary material for: Potential survival benefits of open over laparoscopic radical gastrectomy for gastric cancer patients beyond three years after surgery: result from multicenter in-depth analysis based on propensity matching
Source: Surg Endosc. 2021 Jun 3;36(2):1456–65. doi: 10.1007/s00464-021-08430-0 (PMC8758649; doi:10.1007/s00464-021-08430-0)
Supplement: Supplementary file 10 — Supplementary file10 (DOC 14 kb) [file 464_2021_8430_MOESM10_ESM.doc]

**Supplemental table 4.** Frequencies of causes of recurrence within 5 Years after surgery in patients who underwent ODG or LDG

| Events | ODG (n=461) | LDG (n=461) | P-value |
| --- | --- | --- | --- |
| **Any recurrence** | 137(29.7) | 140(30.1) | 0.286 |
| **Local** | 44(9.5) | 33(7.2) | 0.234 |
| **peritoneum** | 32(6.9) | 46(10.0) | 0.123 |
| **Multiple site** | 13(2.8) | 11(2.4) | 0.837 |
| **Other or uncertain site** | 48(10.4) | 50(10.8) | 0.915 |

Refers only to first-time recurrence, even though patients can have recurrence at multiple times.

Multiple site: includes patients who have recurrence simultaneously in 2 or more metastatic sites, including peritoneum, liver, lung, bone, brain, distant lymph node,or other hematogenous metastatic sites.
