# Supplementary material for: Potential survival benefits of open over laparoscopic radical gastrectomy for gastric cancer patients beyond three years after surgery: result from multicenter in-depth analysis based on propensity matching
Source: Surg Endosc. 2021 Jun 3;36(2):1456–65. doi: 10.1007/s00464-021-08430-0 (PMC8758649; doi:10.1007/s00464-021-08430-0)
Supplement: Supplementary file 11 — Supplementary file11 (DOC 14 kb) [file 464_2021_8430_MOESM11_ESM.doc]

**Supplemental table 5.** Frequencies of causes of recurrence within 5 Years after surgery in cT4a patients who underwent ODG or LDG

| Events | ODG (n=231) | LDG (n=254) | P-value |
| --- | --- | --- | --- |
| **Any recurrence** | 102(44.2) | 105(41.3) | 0.165 |
| **Local** | 34(14.7) | 24(9.4) | 0.092 |
| **peritoneum** | 17(7.4) | 29(11.4) | 0.162 |
| **Multiple site** | 8(3.5) | 10(3.9) | 0.903 |
| **Other or uncertain site** | 43(18.6) | 34(13.4) | 0.136 |

Refers only to first-time recurrence, even though patients can have recurrence at multiple times.

Multiple site: includes patients who have recurrence simultaneously in 2 or more metastatic sites, including peritoneum, liver, lung, bone, brain, distant lymph node,or other hematogenous metastatic sites.
