# Supplementary material for: Potential survival benefits of open over laparoscopic radical gastrectomy for gastric cancer patients beyond three years after surgery: result from multicenter in-depth analysis based on propensity matching
Source: Surg Endosc. 2021 Jun 3;36(2):1456–65. doi: 10.1007/s00464-021-08430-0 (PMC8758649; doi:10.1007/s00464-021-08430-0)
Supplement: Supplementary file 12 — Supplementary file12 (DOC 14 kb) [file 464_2021_8430_MOESM12_ESM.doc]

**Supplemental table 6. Frequencies of causes of recurrence within 5 Years after surgery in cT4a/tumor size ＞5cm patients who underwent ODG or LDG**

| Events | ODG (n=82) | LDG (n=112) | P-value |
| --- | --- | --- | --- |
| **Any recurrence** | 35(42.7) | 60(53.4) | 0.178 |
| **Local** | 13(15.9) | 15(13.4) | 0.682 |
| **peritoneum** | 4(4.9) | 17(15.2) | 0.033 |
| **Multiple site** | 4(4.9) | 6(5.4) | 1.000 |
| **Other or uncertain site** | 14(17.1) | 22(19.6) | 0.711 |

Refers only to first-time recurrence, even though patients can have recurrence at multiple times.

Multiple site: includes patients who have recurrence simultaneously in 2 or more metastatic sites, including peritoneum, liver, lung, bone, brain, distant lymph node,or other hematogenous metastatic sites.
